# Editorial: Lymphoid cells and tumor microenvironment: a functional crosstalk

**DOI:** 10.3389/fmed.2023.1319904

**Published:** 2023-10-30

**Authors:** Selena Mimmi, Sabino Ciavarella, Massimo Gentile, Enrico Iaccino

**Affiliations:** ^1^Department of Clinical and Experimental Medicine, Magna Græcia University of Catanzaro, Catanzaro, Italy; ^2^Hematology and Cell Therapy Unit, IRCCS Istituto Tumori “Giovanni Paolo II”, Bari, Italy; ^3^Department of Hematology and Oncology, Health Agency of Cosenza, Cosenza, Italy; ^4^Department of Pharmacy, Health and Nutritional Science, University of Calabria, Rende, Italy

**Keywords:** tumor microenvironment, B cells, T cells, extracellular vesicles (EVs), exosomes, immune system, hematopoiesis and hematopathology

## Introduction

Normal hematopoiesis as well as cancer represent processes orchestrated by a diverse ensemble of cells of both stromal and immune origin maintaining a delicate balance between physiological stimuli and response to their pathological disruption (1, 2). In recent years, remarkable strides have been made in unraveling the complex interplay between stromal and immune cells and their impact on both physiological and pathological processes including hematopoiesis as well as hematological malignancies (3). This editorial presents a Research Topic of articles that delve into the multifaceted roles of stromal and immune cells in the context of both normal hematopoiesis, solid cancers and lymphoproliferative disorders. Through a comprehensive exploration of the recent research experiences, we aim to shed light on the latest discoveries and emerging concepts that contribute to our understanding of these dynamic cellular interactions. The articles in this series examines fundamental mechanisms through which inflammation and immune cell modulation by infectious agents and tumor cells impact hematopoietic stem cells and their supporting stroma, in turn producing vicious cycles for modulating tumor growth. In particular, most of the reported works explore the role of extracellular vesicles (EVs) as an alternative way of communication between malignant and accessory cells within the tumor microenvironment (TME) (4). EVs are small vehicles of a wide range of molecules, such as proteins, DNA, RNA, miRNAs, and cytokines (5), capable of modifying phenotype or intracellular pathway of target cells, also resulting in pro-survival stimuli for cancer elements (6).

Collectively, these articles provide a snapshot of novel mechanisms in the context of normal hematopoiesis and cancer and aims to inspire further research and therapeutic advancements in the field.

## Overview

### EVs as modulators of tumor survival and progression

It is well known that EVs are secreted by all cell types and play a crucial role in the pathogenesis and progression of several diseases, such as B lymphoproliferative dyscrasias.

Laurenzana et al. focused attention on myeloma multiple-derived EVs (MM-EVs) and how they could negatively influence normal hematopoiesis, acting directly on hematopoietic stem and progenitor cells (HSPCs). In particular, they demonstrated that MM-EVs caused (i) a dose-dependent reduction of HSPCs viability and colony formation, (ii) an increase of stem and early precursors in the S phase, and (iii) an increased expression level of C-X-C motif chemokine receptor type 4 (CXCR4), promoting a tumorigenic phenotype and supporting tumor progression.

Chronic lymphocytic leukemia (CLL) is another B lymphoproliferative disease in which EVs stimulate tumor survival. CLL cells need to be in close contact with the tumor microenvironment to proliferate, and one of the stimuli is represented by the protein tyrosine kinase Lyn, expressed in the malignant and stromal cells. In this contest, de Oliveira et al. demonstrated a positive relationship between Lyn and stroma-derived EVs inasmuch as Lyn is shown to increase CLL-supportive EVs secretion by stromal cell lines as compared to Lyn-knocked down ones.

### Immune microenvironment reprogramming in solid tumors

Hepatoblastoma (HB) is the most common liver malignancy in childhood, with a poor prognosis and lack of effective therapeutic targets. According to Guo et al. the immune microenvironment acts an essential role in the pathogenesis of HB, especially natural killer (NK) cells. They found that the inhibitory receptor KIR2DL is upregulated in HB-derived NK; on the other side, tumor cells express high levels of HLA-C, which interacts with KIR2DL and blocks the antitumor activity of NK. For this reason, developing blockers of the HLA-C/KIR2DL interaction could be used as a new strategy for HB treatment.

### Infections down modulate the immune response

Bone marrow failure (BMF) syndromes are a heterogeneous group of benign hematological diseases characterized by cytopenia, including acquired aplastic anemia, hypoplastic myelodysplastic syndromes, and large granular lymphocyte leukemia. Acquired BMF often emerges after immunosuppressive therapies, however, it is hypotyzed a possible pathogens engagement. Giudice et al. reports that the most represented T cells in BMF are associated with infectious agents as Cytomegalovirus and Mycobacterium tuberculosis. The Authors provide a comprehensive perspective on the main mechanisms for pathogens to trigger autoimmune responses against hematopoietic stem cells, thus leading to acquired BMF.

## Concluding remarks

This Research Topic proposes some pivotal works emphasizing the role of the stromal and immune microenvironment in different solid and hematological tumors, including novel insights on (i) EVs as immunomodulators in two different lymphoproliferative disorders; (ii) the reprogramming capability of TME; and (iii) the role of infections in immune system failure. Overall, the Research Topic gathers new experimental experiences sharing the conclusion that only a deeper mechanistical understanding of TME cellular players and functions could provide future strategies with therapeutically purposes.

## Author contributions

SM: Writing—original draft, Writing—review & editing. SC: Writing—original draft, Writing—review & editing. MG: Writing—original draft, Writing—review & editing. EI: Writing—original draft, Writing—review & editing.
